# Caseous Necrosis of the Mitral Annulus Mimicking a Cardiac Mass: A Rare Presentation

**DOI:** 10.7759/cureus.84146

**Published:** 2025-05-15

**Authors:** Birgurman Singh, Christopher J Murray, Abdulraheem Eniola Hassan, Nirmal J Kaur

**Affiliations:** 1 Internal Medicine, Saint Peter’s University Hospital, New Brunswick, USA; 2 Internal Medicine, Saint Peter’s University Hospital, New Brunswick , USA

**Keywords:** cardiac mri, echocardiography, heart failure, mitral annular calcification, mitral valve, valvular heart disease

## Abstract

Caseous mitral annular calcification (CMAC), a rare variant of mitral annular calcification (MAC), predominantly affects older adults. CMAC represents a very small fraction of MAC, and it features a necrotic core with peripheral calcifications, mimicking neoplasms. Multimodal imaging is essential for diagnosis. We present a case of an 86-year-old hypertensive male patient who presented with exertional dyspnea. Transthoracic echocardiography revealed severe left ventricular hypertrophy, a left cardiac mass, and severe MAC. Cardiac MRI confirmed CMAC (12×14 mm calcified mass). He was managed medically and surveilled with serial echocardiograms. While asymptomatic cases may regress, complications (emboli, valve dysfunction) warrant surgery. Advanced imaging prevents misdiagnosis, guiding intervention. CMAC necessitates multimodal imaging for accurate diagnosis. Conservative management with serial monitoring is appropriate in asymptomatic patients, underscoring the importance of clinician awareness to mitigate complications. Early recognition ensures optimal outcomes in these rarer etiologies.

## Introduction

Mitral annular calcification (MAC) refers to the chronic degeneration of the fibrous ring of the mitral valve, primarily affecting the posterior annulus [1]. Caseous MAC (CMAC) appears as a round, sometimes semilunar, large, echo‐dense, soft mass with central echo-lucencies seen on both transthoracic echocardiography (TTE) and, in particular, transesophageal echocardiography, resembling a periannular mass. It is located at the posterior annular region of the mitral valve, unlike MAC, which usually involves the midbase of the posterior leaflet. Still, it may also involve other segments of the mitral annulus [2]. CMAC is a rare form of degenerative MAC, predominantly affecting older adults with hypertension [3]. Representing 0.6% of MAC cases and up to 0.07% in the general population, CMAC is often under-recognized, with autopsy studies suggesting a higher prevalence [4]. While frequently asymptomatic, it has the potential for significant complications, including severe mitral valve dysfunction and embolization [5].

These masses can grow and infiltrate adjacent territories, such as the myocardium, and cause severe mitral valve dysfunction or consequential outcomes [3]. Diagnosis is challenging, as CMAC’s imaging characteristics evolve unpredictably. A multimodal imaging approach, integrating echocardiography, cardiac CT, and cardiac MRI (CMR), is indispensable for accurate identification. Echocardiography typically reveals a round mass with a central echo-lucent area at the base of the posterior leaflet [4]. Cardiac CT and CMR further aid in distinguishing CMAC from other intracardiac masses by highlighting its unique features, such as peripheral calcifications and a central liquefied core [6].

Peripheral calcifications and avascularity are diagnostic hallmarks, setting CMAC apart from other atrioventricular groove masses like myxomas or aneurysms, which often display vascularity on Doppler or contrast imaging [6]. CMAC is often misdiagnosed as a myocardial abscess, which appears as a mass within the myocardium or annular region, lacks calcifications, and may show systolic blood flow by color Doppler.

Kronzon et al. described three cases of mitral annular masses that, upon excision, were found to be acellular with no evidence of bacterial or fungal growth in cultures [7]. These masses consisted of calcium, cholesterol, and fat, leading the authors to introduce the term "sterile myocardial abscess" to characterize the lesion.

This case highlights the diagnostic challenges and clinical implications of CMAC, emphasizing the need for a high index of suspicion and multimodal imaging to differentiate it from other cardiac pathologies. We present this rare entity to underscore its unique features, management dilemmas, and the importance of individualized patient care.

## Case presentation

An 86-year-old man with a past medical history of hypertension and hyperlipidemia presented to the hospital with worsening exertional shortness of breath. The patient suffered from progressive New York Heart Association (NYHA) Class II-III dyspnea, prompting the emergency room visit. He had no family history of cardiac disease or any murmurs.

On presentation, his vitals were stable: blood pressure was 145/78 mmHg, heart rate was 67 beats per minute, and oxygen saturation was 98% on room air. Physical examination revealed a cachectic but alert and oriented individual. Cardiovascular examination was notable for a 4/6 holosystolic murmur, heard best at the apex but also audible at the left sternal border with no radiation. Bilateral 1+ peripheral edema was present, but there were no signs of respiratory distress, wheezing, or rales on pulmonary examination.

**Figure 1:**
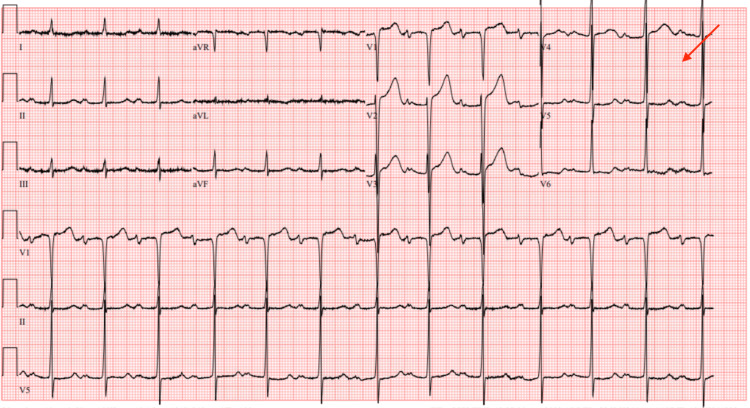
Electrocardiogram showing left atrial enlargement and left ventricular hypertrophy with repolarization abnormalities

Initial evaluation included a 12-lead electrocardiogram, which showed sinus rhythm with possible left atrial enlargement, and left ventricular hypertrophy (LVH) with repolarization abnormalities (Figure 1).

**Figure 2:**
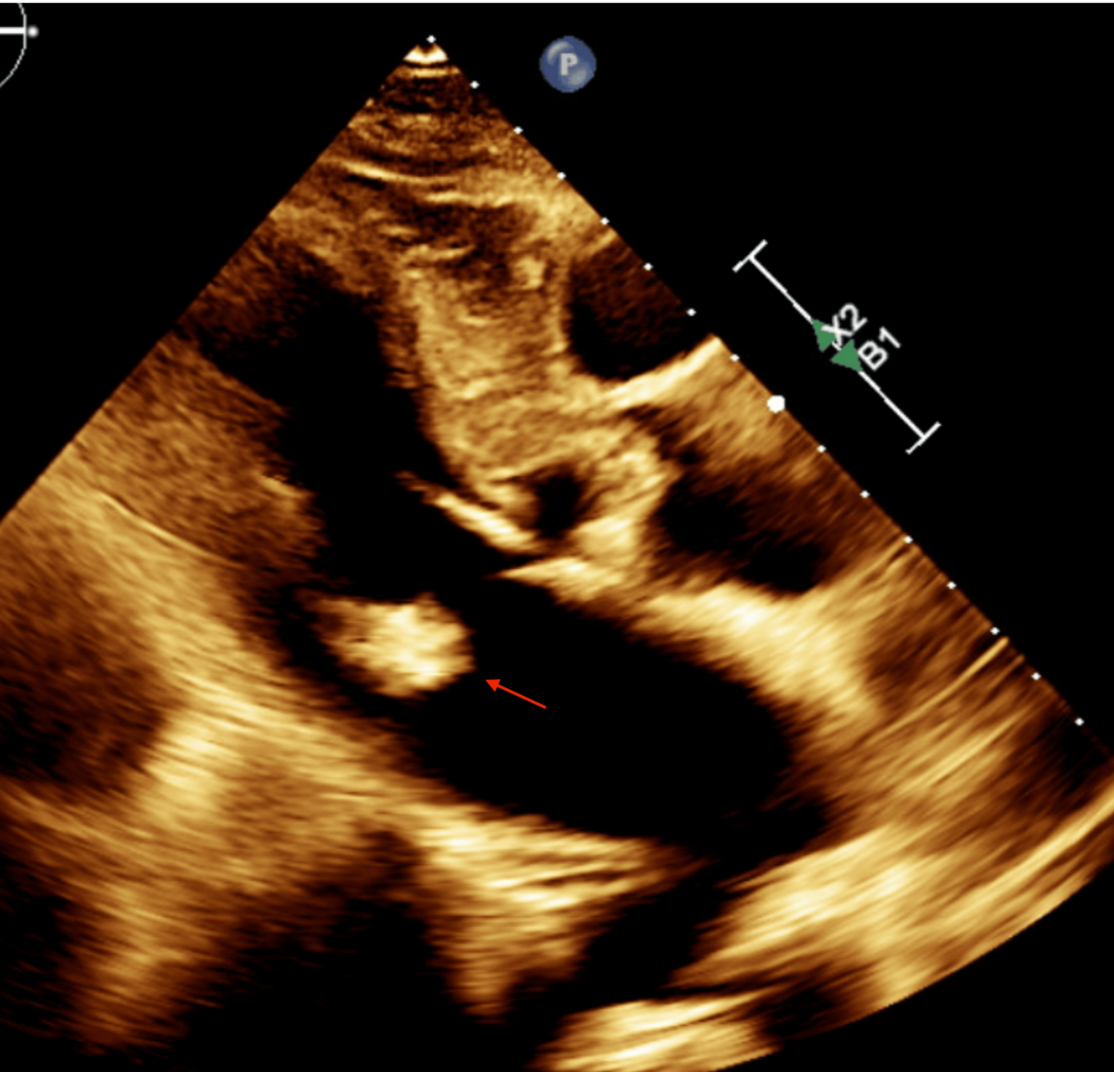
Two‐dimensional echocardiogram showing a calcified mass (red arrow) attached to the mitral annulus

A TTE was subsequently performed, revealing severe asymmetric LVH consistent with hypertrophic cardiomyopathy (HCM), a 1.7 x 1.5 cm mass in the ventricular side of the posterior leaflet, significant MAC predominantly along the inferior and lateral walls, systolic anterior motion of the mitral valve, and mild mitral regurgitation (Figure 2). The left ventricular ejection fraction was preserved at 65%.

**Figure 3:**
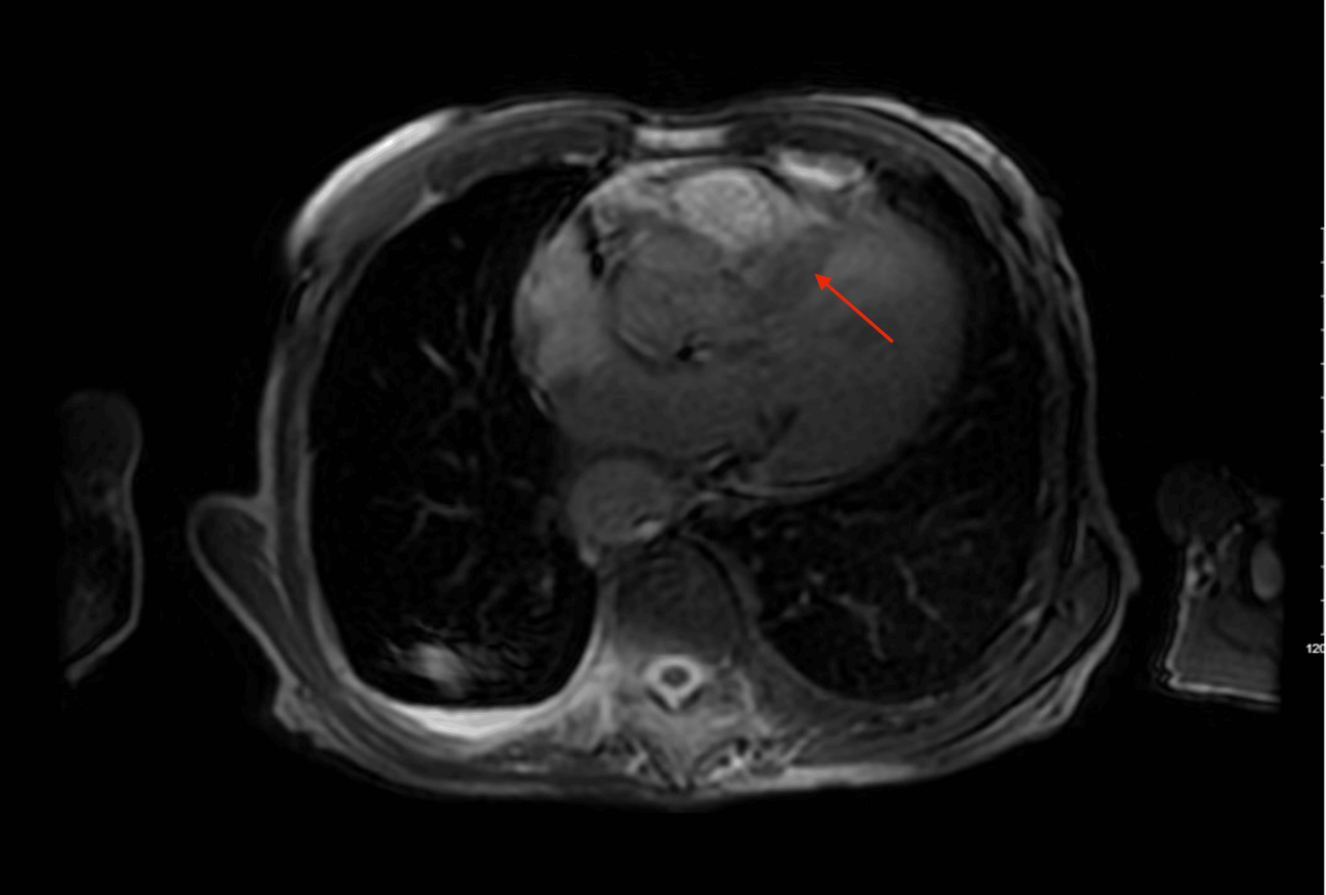
Cardiac MRI (axial view) showing a mass (red arrow) attached to the mitral annulus

**Figure 4:**
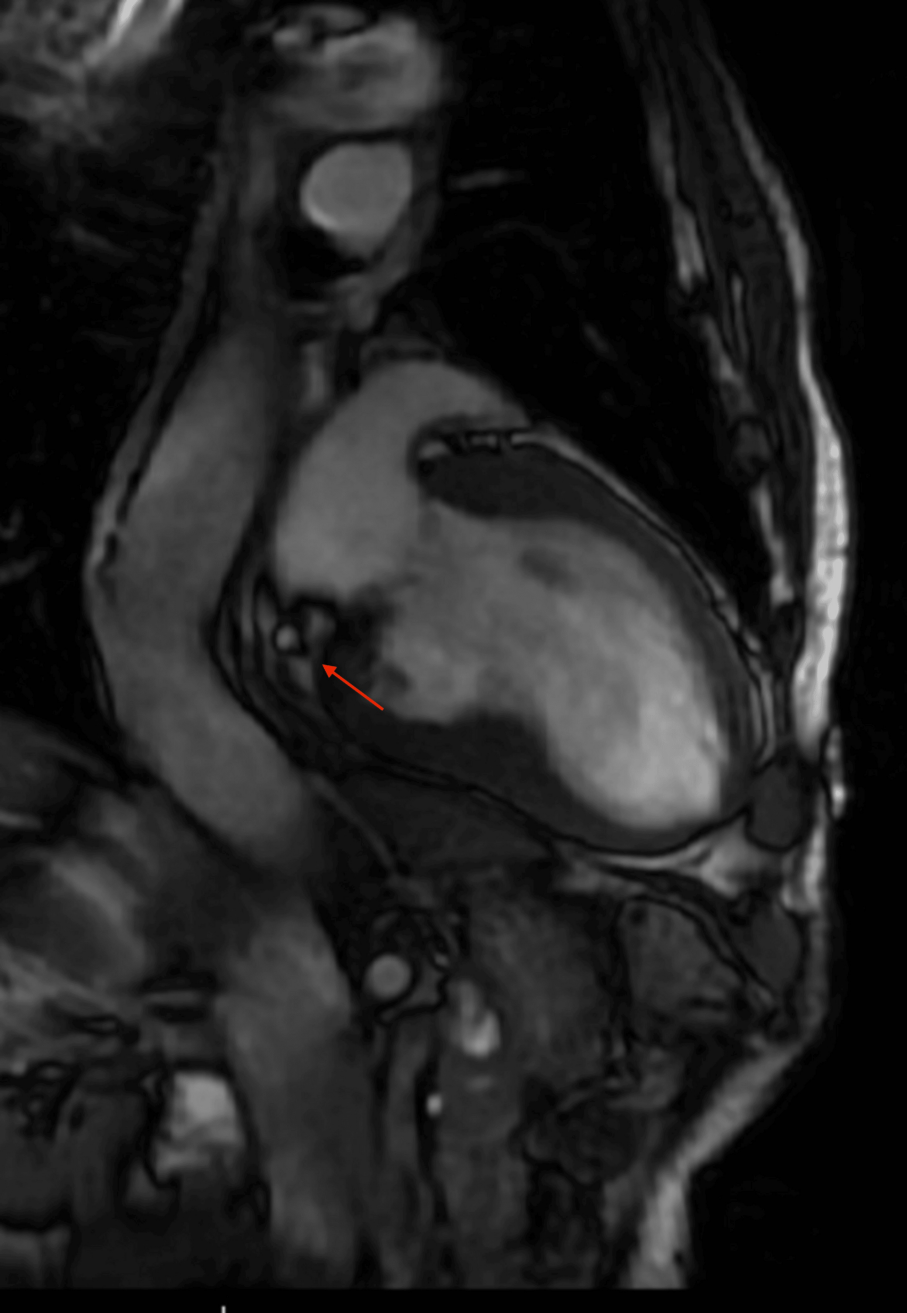
Cardiac MRI (coronal view) showing a mass (red arrow)

**Figure 5:**
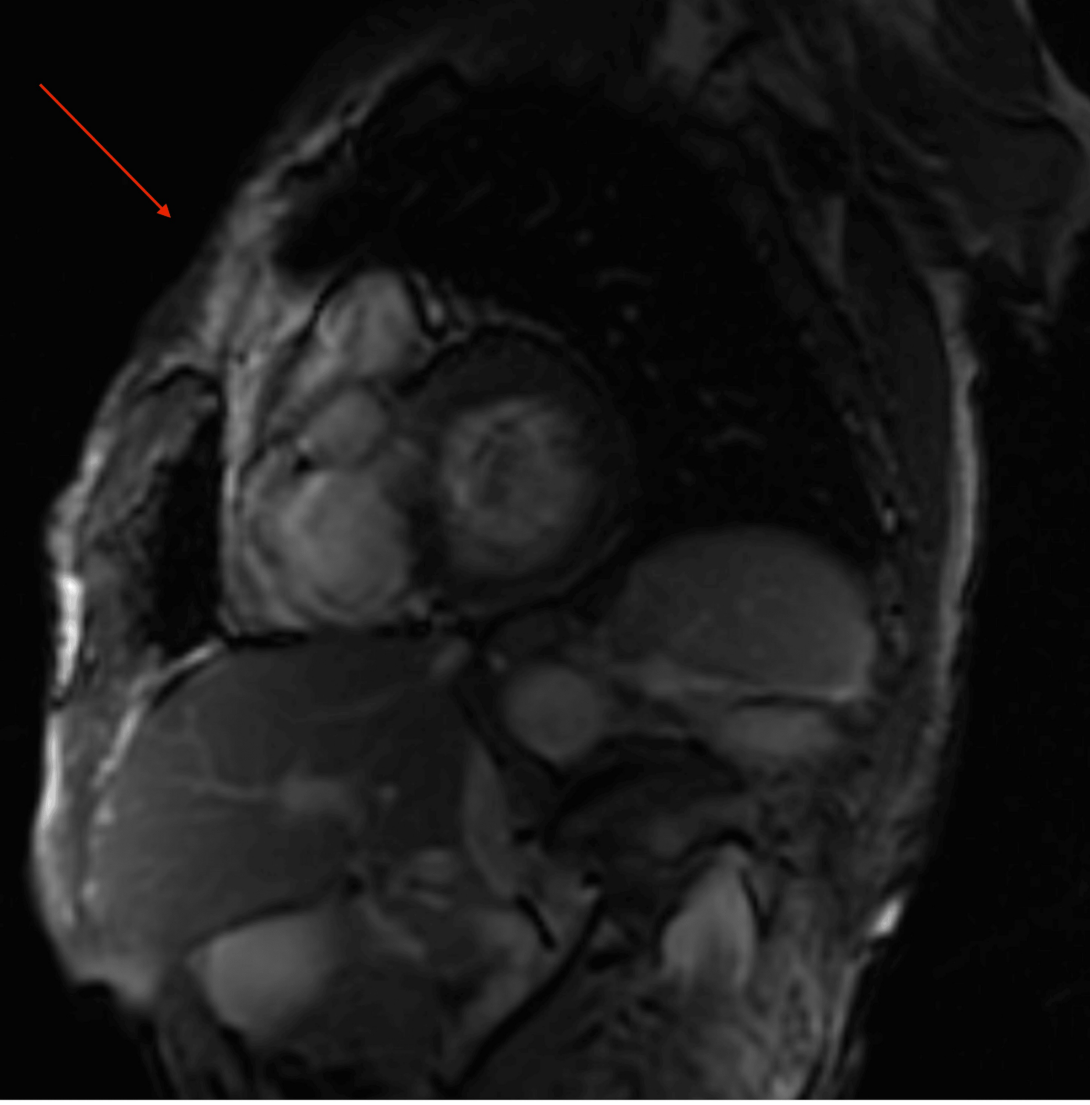
Cardiac MRI (sagittal view) showing a mass (red arrow) attached to the mitral annulus

Given the concerning findings on TTE, a cardiac MRI was obtained for further characterization. The MRI confirmed asymmetric HCM with delayed gadolinium enhancement showing cloud-like mid-wall enhancement in the proximal and distal septum. A 12x14 mm calcified mass on the ventricular side of the posterior mitral leaflet was identified, consistent with CMAC (Figures 3-5).

The patient was managed conservatively; medically, he was maintained on metoprolol, atorvastatin, and torsemide. Family members were advised to undergo screening for HCM. The patient was scheduled for follow-up echocardiography every six to eight months to monitor for disease progression, with particular attention to assessing the size of the mass for stability or regression.

## Discussion

CMAC features a necrotic core of calcium, cholesterol, and fatty acids, accounting for its central echo-lucency on echocardiography and hypodensity on CT imaging. For peri-annular masses, the usual differential diagnoses are thrombus, focal calcification, tumor, abscess, or vegetation. CMAC's avascular nature helps differentiate it from tumours or thrombi. The presence of peripheral calcifications and lack of vascularity are key diagnostic features, distinguishing CMAC from other atrioventricular groove masses such as myxomas or aneurysms, which typically exhibit vascularity on Doppler or contrast imaging [6-8]. 

In the present case, CMR helped diagnose and differentiate CMAC from other masses. It is seen as a “toothpaste-like” consistency as per surgical nomenclature. Physicians must recognise CMAC, which mimics neoplasms and carries distinct clinical implications. Advances in cross-sectional imaging (CT/CMR) have increased its detection, prompting this report to explore CMAC’s diverse imaging features and underscore the role of multimodal imaging in accurate diagnosis. Most cases are asymptomatic and incidentally identified during cardiac imaging. Although CMAC is a benign condition, it can lead to myriad complications, including embolic events, mitral valve dysfunction, stroke, and embolic acute coronary syndrome [9].

In the current case, the patient initially presented with progressively worsening exertional dyspnea, primarily attributed to HCM and uncontrolled hypertension, until echocardiography revealed a cardiac mass, highlighting how CMAC symptoms can mimic other cardiac conditions and complicate diagnosis [10].

Surgical intervention is definitively indicated for CMAC with valvular dysfunction, systemic emboli, or diagnostic uncertainty mimicking malignancy, as conservative management risks catastrophic complications (e.g., embolic stroke, ventricular rupture). Deluca et al.  observed echocardiographic attenuation of CMAC lesions on follow-up imaging, with residual minor calcification [11], while other case reports have described spontaneous resolution [12-14]. These findings support conservative management with serial imaging in asymptomatic patients to monitor for regression, given the condition’s dynamic and potentially self-limiting nature.

The management of CMAC usually depends on the clinical presentation and any associated complications. In the present case, the calcified mass was identified incidentally, and the patient remained asymptomatic without any complications from it. Conservative management was appropriate, with regular follow-ups for any progression in symptoms or the mass.

## Conclusions

This case highlights the complex nature of CMAC, which is a rare but important differential for peri-annular masses and requires high clinical suspicion and multimodality imaging for accurate diagnosis. Clinicians' awareness of CMAC and the use of advanced imaging is crucial to prevent misdiagnosis of valvular vegetation, abscesses, or tumors. It is, therefore, prudent to continue dedicated follow-up with serial imaging, ensuring a good long-term outcome.
